# Sun Exposure and Usage of Sun Protection: Knowledge, Perception and Practice among University Students

**DOI:** 10.21315/mjms2024.31.2.19

**Published:** 2024-04-23

**Authors:** Sadeel A. Shanshal, Shahad M. Khaleel, Sawsan H. Hammoodi

**Affiliations:** 1 College of Pharmacy, Department of Clinical Pharmacy, University of Mosul, Mosul, Iraq; 2 College of Pharmacy, Department of Pharmacology and Toxicology, University of Mosul, Mosul, Iraq; 3 College of Pharmacy, Department of Pharmaceutical Chemistry, University of Mosul, Mosul, Iraq

**Keywords:** sun exposure, skin, sunscreen, skin protection

## Abstract

**Background:**

Exposure to the sun is vital for the body but is associated with problem ranging from pigmentation to cancer. Therefore, knowledge about protective measures is critical. This study aims to assess undergraduate students’ knowledge, perception and practices towards the adverse effects of sun exposure and the usage of sun-protective measures.

**Methods:**

A cross-sectional study was conducted between 1 September 2022 and 20 September 2022 using a web-based questionnaire. Undergraduate students from medical and non-medical colleges in Iraqi universities participated in this study. Descriptive and inferential statistics and logistic regression were performed to analyse the data.

**Results:**

A total of 655 students participated in this study. The knowledge level of more than one-half of the students (53%) was inadequate. Approximately three-quarters of the study sample used sunscreens products. Skin type was the main factor in choosing the appropriate sunscreen product. Media and advice from specialists were the main factors affecting participants’ usage of sunscreens. Gender (females), college (medical), year(s) of study (sixth), residence (urban), daily sun exposure (1 h–3 h) and less-than-normal vitamin D levels were found to be significantly associated with better knowledge (*P*-value < 0.05). Age, gender, being a student in medical colleges and not knowing vitamin D levels were found to be significant predictors of participants’ knowledge.

**Conclusion:**

The low level of knowledge reported among the participants’ calls for more attention from health authorities in educating the public about the harmful effects of sun exposure and the importance of adopting protective measures.

## Introduction

Excessive and unprotected exposure to sun can lead to many skin problems and diseases (1). The deleterious effects of sun exposure range from simple sunburn to severe skin cancer (2). Prolonged exposure to ultraviolet rays is the main cause of different types of skin cancer (3, 4). The incidence of skin cancer has greatly increased in the last decade, which has mainly been attributed to the increase in time spent in leisure and outdoor activities (4).

In contrast, exposure to sunlight is vital for vitamin D synthesis and boosts vitamin D levels; hence, vitamin D is known as the sunshine vitamin (5). Therefore, reduced exposure to sunlight is the leading cause of vitamin D deficiency (6). Many diseases are linked to vitamin D deficiency, such as coronary artery disease, hypertension, diabetes mellitus, rheumatoid arthritis and cancer (7, 8). Therefore, understanding the harmful effects of extended and limited sunlight exposure should be among the healthcare prevention strategies (4).

Adopting appropriate protective measures can aid in preventing the adverse effects of sun exposure while gaining its benefits; these measures include using sunscreen products, seeking shade, avoiding outdoors activities during peak sunlight time, and wearing hats, sunglasses and protective clothing (4, 9–12). Disappointingly, adherence to these protective measures is low and many obstacles may hinder individuals from adhering to these beneficial practices, including inadequate knowledge, misconceptions, inappropriate usage of sunscreen products, inability to start behavioural modifications and other factors such as shortage of time or additional costs (10).

Sunscreens products contain different active ingredients that act as filters to reflect and/or absorb radiation present in sunlight; these active ingredients vary from organic compounds to mineral elements (13, 14). At present, many available sunscreen products have different sun protection factor (SPF) values, which is a measure of the sunscreen product’s ability to protect against sunburn (13).

Adequate knowledge and protective behaviour against the harmful effects of sun exposure are considered critical aspects in preventing many skin problems. Increasing the knowledge and awareness, and encouraging the use of protective measures in the population can help reduce the negative effects of harmful exposures (10, 15).

Several studies have been performed in different countries to assess individuals’ awareness regarding the adverse effects of sun exposure on the skin and the appropriate practices to prevent these effects. Examples include a cross-sectional study conducted by Almuqati et al. (11) among students of nonmedical colleges in Saudi Arabia to assess knowledge, attitude and practice toward sun exposure. The authors found that, despite the good level of participants’ knowledge, sunscreen product usage remained low. They also established that the feeling of discomfort was the primary reason behind the disuse of sun protection products. In another study conducted in Saudi Arabia and Bahrain (4), the participants were found to have adequate knowledge and were aware about the adverse effects of sun exposure, but their level of commitment to protective measures was not optimal. In India, Yashovardhana et al. (9) revealed that Indian youth lacked adequate knowledge about the adverse consequences of sun exposure. This lack of knowledge was suggested by the authors as the reason why many Indians were suffering from various skin problems related to sun exposure without any complainant, since such problems are not lethal.

Published data regarding the knowledge of Iraqi society regarding sun exposure and protection measures are limited. For this reason, this study was conducted to evaluate the knowledge, perception, and practice of Iraqi university students about the adverse effects of sun exposure and the usage of sunscreen products.

## Methods

A cross-sectional design with convenience sampling was applied and the study was conducted among undergraduate university students at Mosul, Iraq between 1 September 2022 and 20 September 2022. The inclusion criteria were being an undergraduate student at Iraqi universities. According to the Iraqi Central Statistical Organization, approximately 850,000 undergraduate students in the Iraqi universities in 2020 (https://www.cosit.gov.iq/ar/2013-02-25-07-39-31). Using this number as the population size, the Raosoft sample size calculator (http://www.raosoft.com/samplesize.html) estimated the minimum sample size to be 384 students.

Data for this study were gathered using a web-based questionnaire and a Google Forms design was adopted for this purpose. The Google Forms link was distributed online to social media groups for university students (using Facebook, Instagram, Messenger and WhatsApp) either by contacting groups’ administrators or directly sending the link to participants. An online link was available during data collection period, which lasted about 20 days. In addition, the participants were reminded twice to prompt their responses to the questionnaire. The Google Forms setting was adjusted to only one response to prevent participants from answering more than once.

The questionnaire used in this work was based on other studies (10, 11, 14, 16). It consisted of 42 questions that were divided into four sections. The sociodemographic characteristics such as sex, age, college, year of study, residence (urban or rural), daily sun exposure and vitamin D level (if the participant recently measured it) were assessed in the first section. The second, third and fourth sections were used to assess university students’ knowledge regarding adverse effects of sun exposure (18 questions), perception toward using protective measures (6 questions) and practice of using sunscreens and other protective strategies (11 questions), respectively. Before conducting the survey, it was translated back into Arabic (17–19). In summary, the original English questions were first forward-translated to Arabic by two independent translators. The two Arabic versions were back-translated to English by a third translator and the differences between the new version and the original English version were accounted for by a discussion between the translator and the researcher. The final Arabic version was taken from there.

After completing the translation, the final Arabic version was available for validity and reliability testing. Validation was performed using content validity, in which the questionnaire was assessed by experts in clinical pharmacy (two academics with a PhD in Clinical Pharmacy from the Department of Clinical Pharmacy, College of Pharmacy, University of Mosul). The two experts provided some suggestions on the choice of certain questions and the wording used in the other questions. Their comments and suggestions were appropriately taken, and the questionnaire was modified accordingly. Reliability was measured using Cronbach’s alpha test (the sample of 30 participants used for the reliability testing was excluded from the final analysis).

A total knowledge score was calculated as follows: 1 point was given for each correct answer and 0 points were given for both incorrect and “I do not know” answers. Therefore, the total score could range from 0 to 18. The median split method was used to categorise participants into two groups based on their knowledge scores (having adequate or inadequate knowledge).

The aims of the study were explained to the participants in the first page of the questionnaire. Participation was completely voluntary as the participants were asked to tick in the agreement of participation on the Google Forms of the questionnaire before participating in the study.

After link distribution, the responses were collected by using Microsoft Excel sheet and the obtained data were processed, coded and analysed using IBM SPSS for Windows version 28.0 (Armonk, NY: IBM Corp.). Descriptive statistics were used to present the different sociodemographic characteristics and inferential statistics were used to study the differences between variables. Logistic regression analysis was performed to predict the effects of the sociodemographic characteristics of the study sample on the likelihood of falling within any of the two knowledge categories (adequate and inadequate). For this purpose, a binary model was constructed, and univariate and multivariate analyses were performed. A *P*-value of less than 0.05 was considered statistically significant.

## Results

A total of 687 participants filled out the questionnaire, among whom 32 were not able to complete all the questions. The final study sample comprised a total of 655 participants, with 193 (29.5%) males and 462 (70.5%) females, with approximately half (333, 50.8%) aged between 21 years old and 23 years old. The sociodemographic characteristics are presented in Table 1. More than half (366, 55.9%) of the students were from nonmedical colleges, and 192 (29.3%) were in their fourth year. Majority of the participants (546, 83.4%) lived in urban areas, and daily sun exposure was less than 1 h for 259 (39.5%) of them. When the participants were asked about their vitamin D level, 58.5% declared that they had not recently checked their vitamin D level, 26.3% answered that their vitamin D level was less than normal, and only 14.7% mentioned that their level was higher than the normal range.

The Cronbach’s alphas for knowledge, perception and practice sections in the questionnaire employed in this study were 0.792, 0.723 and 0.730, respectively, indicating the reliability of the questionnaire.

Table 2 summarises the responses of the participating students to the questions in the knowledge and perception sections. In the first four questions of the knowledge section related to the damaging effects of sun exposure, the participants knew the correct answer mostly for skin pigmentation (90.8%), followed by burns (85.3%), accelerating skin aging (68.7%) and skin cancer (58.8%). The fact that the sun is most harmful between 10:00 a.m. and 2:00 p.m. was correctly reported by 71.8% of the participants. About three-quarters (76.6%) of the participants knew about the relationship between sun exposure and vitamin D levels. A similar percentage (74.3%) correctly reported that sun protection products can shield the skin. Regarding the adverse effects of using sunscreen products, only 27.2% of the students knew that sunscreens may cause acne and 46.1% knew that skin allergies are possible side effect from using sunscreens. Approximately one-third (32.8%) of the participants incorrectly thought that sunscreens may whiten the skin and only 28.2% knew that sunscreens do not affect vitamin D levels. Approximately half of the participants (51.2%) accurately answered that sunscreen products are required even on a cloudy day and slightly less than half (42.1%) were aware that products with higher SPF values provide better protection. One-quarter of the participants (24.6%) incorrectly answered that once-a-day application of sunscreen is effective whereas 59.7% accurately understood that sunscreen products should be reapplied every 2 h even on cloudy days. Considerable percentages (44.8% and 53.9%) falsely thought that sunscreen is the only protection against the harmful effects of the sun and that staying in shade and using umbrellas cannot shield against the sun, respectively. A good majority (71%) of the participants knew accurately that applying sunscreen only on the face is not sufficient.

Regarding responses to perception questions, 83.1% of the students reported advising others about using protection against the damaging effects of the sun and only 37.1% believed that such protection is difficult. About three-quarters of the participants (73.2%) thought that damage to the skin brought about by the sun can be avoided and a similar fraction (78.5%) assumed that clothes play a role in protecting the skin against the sun. However, only 48.4% of the participants thought that staying in the shade is an efficient strategy to get protection from the sun. Finally, 67.5% of the participants stated that they encourage parents to use sunscreen products on their children (Table 2).

**Figure 1:**
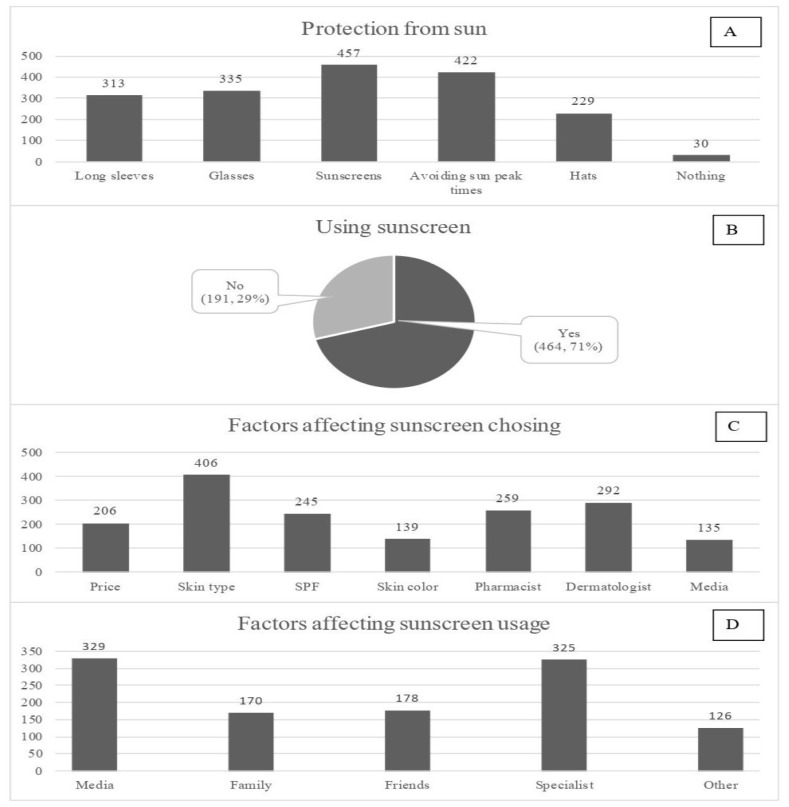
A. Approaches used by the participants to obtain protection from the sun (more than one response was permitted per participant), B. Participants who use sunscreen products, C. Factors affecting the choice of sunscreen products (more than one response was permitted per participant), and D. Factors affecting the usage of sunscreen products (more than one response was permitted per participant)

Applying sunscreen products was the main approach to be used by participants to obtain protection from the sun (457 participants) followed by avoiding going out when the sun is at its peak (422 participants) (Figure 1A). Moreover, 71% of the participants reported that they use sunscreen products (Figure 1B) and when the remaining 29% were asked why they do not use sunscreen products, 27% of them mentioned having no time, and 26% reported ‘other reasons’ for their disuse of sunscreen products. Comparable percentages of students at 22% and 20% declared that feeling discomfort and ignorance about sunscreen efficacy were the reasons for not using these products, respectively, and only 5% mentioned that they did not use sunscreen products because they thought that these products are not compatible with cosmetics and other skin products. The majority of the participants (406) considered skin type as the basis for choosing a sunscreen product, followed by advice from a dermatologist (292 participants) or a pharmacist (259 participants), whereas media was the least reported factor (135 participants) (Figure 1C). When the students were asked about the factors that could affect their use of sunscreen products, social media came first (329 participants), followed by healthcare specialist advice (325 participants), as shown in Figure 1D.

The responses of the participants to the remaining questions in the practice section are presented in Table 3. Regarding when to use sunscreens, 58.2% of the participants answered, ‘Outdoors only.’ Avoiding skin burns and preventing skin pigmentation were the main reasons for using sunscreens as reported by 73.7% and 69.6% of the participants, respectively. Sunscreen products with SPF values between 30 and 50 were preferred by most participants at 23.8%. More than half of the participants (53.7%) stated that they apply sunscreen 15 min–30 min before going out but only 15.3% reapplied sunscreen every 2 h.

The final question in the practice section asked the participants about the area(s) of their body on which they usually apply sunscreen. Slightly more than one-third of the participants (35.7%) reported face and hands, and almost one-third (33.7%) answered face only. Only 32 participants (4.9%) reported applying sunscreen on the face, hands, neck and legs. The remaining 25.6% stated applying sunscreen on different combinations of the face, hands, neck and legs and other than those mentioned above.

The score for the knowledge section ranged between 0 and 18, with a mean of 10.04 (SD 3.13). The median was 10 and was used for the categorisation of the participants into those having adequate knowledge, whose score was more than 10, and those with inadequate knowledge, whose score was 10 or less. The mean score for those with adequate knowledge was 12.74 ± 1.53, which constituted approximately 47% (307) of the participants. In total, 348 students (53%) had inadequate knowledge and scored an average of 7.67 ± 2.08 in the knowledge section.

The differences in the mean score of the knowledge section in the different classes of the categorical variables are presented in Table 4. Apart from the age groups, significant differences in the mean knowledge score were found, with the highest scores in females, students in medical colleges, sixth year students, those living in urban areas, those with 1 h–3 h of daily sun exposure and those with less-than-normal vitamin D levels.

Logistic regression analysis was performed to test the effect of the sociodemographic characteristics presented in Table 1 on the odds of being adequately or inadequately knowledgeable about sun exposure and the usage of sun protection. The resulting model proved to be statistically significant (chi-square = 174.040, *P*-value < 0.001). Participants aged more than 23 years old showed a reduction of 41% in the odds of having adequate knowledge compared to those between 18 years old and 20 years old, and when the other independent variables were included in the analysis, the likelihood of this reduction increased to approximately 50%. Females were five times likelier to have adequate knowledge than males in both univariate and multivariate analyses. In contrast, participants from nonmedical colleges were less likely to be adequately knowledgeable. Participants in both the fifth and sixth years of study were predicted to be three times likelier to have adequate knowledge compared to first year participants in the univariate analysis only, and this influence was not observed when other variables were included. Residence is a significant predictor only when considered on its own, with those living in rural areas having approximately 47% less chance of being adequately knowledgeable compared to those living in cities. Participants who did not know their vitamin D levels were shown to be less likely to have adequate knowledge in both univariate and multivariate analyses. A detailed description of these different odds ratios, along with their levels of significance, is presented in Table 5.

## Discussion

Despite the benefits of sun exposure being the main source of vitamin D in the body, it has many deleterious effects on the skin, ranging from simple skin burns to different skin cancers. Fortunately, these negative effects can be prevented by adopting protective measures, such as using sunscreen products, avoiding outdoor activities at sunlight peak times, and wearing hats, long sleeves, and sunglasses. Proper behaviour regarding sun exposure, combined with the application of protective measures, can prevent up to 80% of skin cancer cases (20). This study evaluated the awareness of university students towards sun exposure and protection; this section of society was chosen because using sun-protective measures in such age groups has proven to be effective in preventing skin problems (11). More than half of the participants had inadequate knowledge about the threats of sun exposure, and the knowledge scores varied with different variables, such as gender, college, year of study, residence and daily exposure to the sun.

The female participants in this study were more represented than their male counterparts, which was similar to a study conducted in Bosnia and Herzegovina, where 64.3% of the participants were females (20). In addition, this latter study had a similar participant distribution between medical and nonmedical colleges (50% in each) to our study.

In the current study, the question that reported the most correct answers in the knowledge section was about the effect of sun exposure on skin colour, with more than 90% correct answers, which was in parallel to the results reported by Yurtseven et al. (21). Approximately 80% of the participants in the study by Al-mutairi et al. (22) knew about the relationship between sun exposure and vitamin D levels, which was comparable to the results obtained in the present study.

Compared to less than half of the participants in the current study, approximately three-quarters of the participants in the study by Awadh et al. (14) knew the correct answer for the question regarding the relation between the SPF value and the effectiveness of protection. This finding could be related to the sample of the two studies, where, in the Awadh et al. (14) study, only pharmacy and medical students were included, whereas in our study, students from nonmedical colleges were permitted to participate.

As revealed in this study, the most common approach for protection against the deleterious effects of the sun was found to be the use of sunscreen in the studies conducted by Iglesias-Puzas et al. in Spain (23) and by Al-mutairi et al. in Kuwait (22). This finding could be explained on the basis of the ease of the sunscreen application, advertisements of sunscreen on different media, and the fact that the application of these products does not hinder daily activities (24). However, some studies reported different results; for example, in the study on university students in Turkey by Yurtseven et al. (21), staying in shade was the preferred method of protection against the sun.

Apart from sunscreen application, our results showed that avoiding sun exposure during peak times was the preferred protection method, followed by wearing sunglasses, long sleeves and hats, respectively. This was slightly different from a study conducted on university students in Poland, where sunglasses came immediately after sunscreen, followed by wearing hats and avoiding peak times of sun exposure (25). This difference in the order of protective measures may be attributed to the difference in the intensity of sunlight between Iraq and Poland. In Iraq, extreme heat may contribute to people’s desire to avoid direct sun exposure, whereas in Poland, the heat may not reach extreme levels.

The Polish study reported comparable results to the current work in terms of the practice of the participants with sunscreen application frequency, where approximately two-thirds of the participants in the two studies admitted to applying sunscreen only once; in addition, regarding the time of application, 29% and 26% of the students in the Polish study and this work reported applying sunscreen very shortly or immediately before going out, respectively (25).

Although one-quarter of the participants in the current study practiced the application of sunscreen immediately before going outdoors, more than one-half reported the appropriate practice of waiting 15 min–30 min after application and before going out. This finding was parallel to the study by Alzahrani et al. (26) which also reported a similar proportion to the current study of the participants who believe that sunscreen should be reapplied. In the current study, a difference was observed between those who knew that sunscreen should be reapplied even on cloudy days (59.7%) and those who practiced reapplication (15.3%). This result could be attributed to the ease of the reapplication process and the reluctance of participants to take the product and apply it when they are outside.

Skin type was the main factor in choosing the sunscreen in the current study, whereas SPF value was the main factor in the study conducted by Awadh et al. (14). In this study, for the participants who did not use sunscreens, having no time was their main reason, which was similar to that in (26) and (27). In the current study and in the study of Awadh et al. (14), media was the factor that had the most influence among the participants on sunscreen usage; however, our study differed in that many participants showed willingness to advise others regarding sun protection (83% compared to 62%) in (14). Approximately two-thirds of the participants in the current study reported that they encourage parents to use sunscreen products on their children. This finding was much higher than in the Malaysian study (14) where only one-third of the participants would have this attitude.

In a Saudi Arabian study that included medical staff, the mean knowledge score was 6.85 out of 18 (26), which was well below the score reported in the current study (10.04). In the present study, younger participants scored better than older ones in the knowledge section, which might explain the difference in the knowledge scores between the two studies. In the Saudi Arabian study, the mean age of the participants was 25.31 years old (26) which is older than the majority of our participants (approximately 80% are younger than 23 years old).

In contrast to our study, which showed that females had significantly higher knowledge scores, the study in Bosnia and Herzegovina showed no difference in these scores between the two genders (20) as did the study in Turkey (21). However, in the studies conducted by Al-mutairi et al. (22) and Awadh et al. (14), a similar pattern of females having better knowledge was reported. This could be more plausible, as females are known to pay more attention to skin care and protection than males.

More than 80% of the participating students in a Brazilian study (27) had satisfactory knowledge scores which is much higher than the 47% reported in this study; however, the two studies shared the finding that participants from medical colleges have better knowledge than those from non-medical colleges. Medical participants were also reported to have better knowledge in a Polish study (25).

Administering the questionnaire online may be regarded as a limitation to this study as it eliminated face-to-face contact between the researcher and the participants. However, online surveys allow for wider reachability and faster response times than face-to-face interviews. The cross-sectional design of the study could also be considered a limitation; however, the aim was to evaluate the level of knowledge among the participants and to derive conclusions and suggestions that will improve the behaviour of the population regarding sun exposure and protection.

## Conclusion

Although more than 70% of the students reported using sunscreen products, less than half of the study sample had adequate knowledge regarding the adverse effects of sun exposure and protective measures. This knowledge is even worse in male participants, participants from non-medical colleges, participants from first, second and third years of study, participants living in rural areas and those who do not know their vitamin D levels. This low level of knowledge among this section of society about such an important aspect of general health should be considered by health authorities to minimise the risk of skin cancer. Educational campaigns through posters, directed videos on social media, and in-campus seminars and lectures can help accomplish this task.

## Figures and Tables

**Table 1:** Sociodemographic characteristics of the study sample

Variables	Frequency (%)
Age (years old)	
18–20	194 (29.7)
21–23	333 (50.8)
> 23	128 (19.5)
Gender	
Male	193 (29.5)
Female	462 (70.5)
College	
Medical	289 (44.1)
Non-medical	366 (55.9)
Year of study	
First	39 (6.0)
Second	171 (26.1)
Third	124 (18.9)
Fourth	192 (29.3)
Fifth	108 (16.5)
Sixth	21 (3.2)
Residence	
Urban	546 (83.4)
Rural	109 (16.6)
Daily sun exposure (hour)	
< 1	259 (39.5)
1–3	219 (33.4)
> 3	177 (27.0)
Vitamin D level	
< Normal level	173 (26.3)
Within normal level	96 (14.7)
> Normal level	3 (0.5)
Do not know	383 (58.5)

**Table 2:** Responses of the participants to questions in the knowledge and perception sections

Questions		Answers		

		Yes *n* (%)	No *n* (%)	Do not know *n* (%)
	Knowledge			

Q1	Sun exposure causes burns	559 (85.3) a	66 (10.1)	30 (4.6)
Q2	Sun exposure causes skin cancer	385 (58.8) a	80 (12.2)	190 (29.0)
Q3	Sun exposure accelerates skin aging	450 (68.7) a	66 (10.1)	139 (21.2)
Q4	Sun exposure causes skin pigmentation	595 (90.8) a	25 (3.9)	35 (5.3)
Q5	Sun is most harmful between 10:00 a.m. and 2:00 p.m.	470 (71.8) a	84 (12.8)	101 (15.4)
Q6	Sun exposure affects vitamin D level	502 (76.6) a	32 (4.9)	121 (18.5)
Q7	Sun protection products provide shield for skin	487 (74.3) a	83 (12.7)	85 (13.0)
Q8	Sunscreen causes acne	168 (27.2) a	187 (28.5)	290 (44.3)
Q9	Sunscreen causes skin allergy	302 (46.1) a	120 (18.3)	233 (35.6)
Q10	Sunscreen causes skin whitening	215 (32.8)	206 (31.5) a	234 (35.7)
Q11	Sunscreen negatively affects vitamin D level	117 (17.9)	185 (28.2) a	353 (53.9)
Q12	Sunscreen is required on cloudy days	335 (51.2) a	230 (35.1)	90 (13.7)
Q13	Higher SPF provides better protection	276 (42.1) a	83 (12.7)	296 (45.2)
Q14	Applying sunscreen once a day is effective	161 (24.6)	378 (57.7) a	116 (17.7)
Q15	Sunscreen is the only way to protect against sun	294 (44.8)	263 (40.2) a	98 (15.0)
Q16	Shade and umbrellas cannot protect from the sun	353 (53.9)	152 (23.2) a	150 (22.9)
Q17	Sunscreen needs reapplication even on cloudy days	391 (59.7) a	137 (20.9)	127 (19.4)
Q18	Applying sunscreen only on the face is enough	112 (17.1)	465 (71.0) a	78 (11.9)

	Perception			

Q1	Advise others for sun protection	544 (83.1)	73 (11.1)	38 (5.8)
Q2	Protecting skin from sun is difficult	243 (37.1)	370 (56.5)	42 (6.4)
Q3	Sun damage to the skin can be avoided	479 (73.2)	111 (16.9)	65 (9.9)
Q4	Clothes play a role in protection against the sun	514 (78.5)	72 (11.0)	69 (10.5)
Q5	Staying in shade is effective protection from the sun	317 (48.4)	268 (40.9)	70 (10.7)
Q6	Encourage parents to apply sunscreen on their children	442 (67.5)	131 (20.0)	82 (12.5)

Note:

a correct answer

**Table 3:** Responses of the participants to the questions in the practice section

Variable	Frequency (%)
When to use sunscreen	
Outdoors and indoors	76 (11.6)
Outdoors only	381 (58.2)
Gardens only	35 (5.3)
Swimming only	10 (1.5)
Not using	153 (23.4)
Why use sunscreen*	
Whiter	122 (18.6)
Prevent pigmentation	456 (69.6)
Avoid burning	483 (73.7)
Avoid wrinkles	231 (35.3)
Old age	217 (33.1)
Colour change	336 (51.3)
Avoid spot	78 (11.9)
Avoid cancer	220 (33.6)
SPF value	
< 30	20 (3.1)
30–50	156 (23.8)
50–70	138 (21.1)
> 70	19 (2.9)
Do not know	322 (49.2)
When to apply sunscreen	
Immediately before	172 (26.3)
15 min–30 min	352 (53.7)
30 min–60 min	74 (11.3)
10 min after	57 (8.7)
Frequency of reapplying sunscreen	
Every 2 h	100 (15.3)
Every 3 h	57 (8.7)
Every 4 h	103 (15.7)
No need to reapply	395 (60.3)

Note:

* More than one response was permitted per student for this question

**Table 4:** Differences in knowledge scores between different groups of categorical variables

Variable	Frequency	Knowledge mean ± SD	*P*-value
Age* (years old)			0.077
18–20	194	10.25 ± 2.92	
21–23	333	10.14 ± 3.28	
> 23	128	9.49 ± 2.98	
Gender**			< 0.001§
Male	193	8.19 ± 2.97	
Female	462	10.82 ± 2.85	
College**			< 0.001§
Medical	289	11.39 ± 2.71	
Non-medical	366	8.98 ± 3.02	
Year of study*			< 0.001§
First	39	9.51 ± 3.00	
Second	171	9.36 ± 2.98	
Third	124	9.14 ± 3.13	
Fourth	192	10.07 ± 3.04	
Fifth	108	11.92 ± 2.76	
Sixth	21	12.14 ± 2.19	
Residence**			< 0.001§
Urban	546	10.24 ± 3.06	
Rural	109	9.08 ± 3.30	
Daily sun exposure* (hour)			0.031§
< 1	259	9.95 ± 3.06	
1–3	219	10.47 ± 3.09	
> 3	177	9.66 ± 3.21	
Vitamin D level*			< 0.001§
< Normal	173	11.24 ± 2.79	
Within normal	96	10.29 ± 2.74	
> Normal	3	10.00 ± 2.00	
Do not know	383	9.44 ± 3.21	

Notes:

* One-way ANOVA;

** independent samples *t*-test;

§ significant results

**Table 5:** Univariate and multivariate logistic regression models for having adequate or inadequate knowledge about sun exposure and the use of sun protection

Variable	Univariate logistic regression				Multivariate logistic regression			

	*P-*value	OR	95% CI		*P-*value	AOR	95% CI	
	
			Lower	Upper			Lower	Upper
Age (years old)								
18–20*	-	-	-	-	-	-	-	-
21–23	0.631	0.917	0.643	1.307	0.047§	0.638	0.409	0.994
> 23	0.022§	0.588	0.373	0.927	0.016§	0.498	0.282	0.877
Gender								
Male*	-	-	-	-	-	-	-	-
Female	< 0.001§	5.237	3.532	7.766	< 0.001§	5.311	3.39	8.305
College								
Medical*	-	-	-	-	-	-	-	-
Non-medical	< 0.001§	0.266	0.192	0.368	< 0.001§	0.386	0.257	0.581
Class (year)								
First*	-	-	-	-	-	-	-	-
Second	0.395	0.736	0.364	1.491	0.104	0.504	0.221	1.152
Third	0.526	0.790	0.381	1.638	0.247	0.600	0.253	1.425
Fourth	0.798	1.095	0.547	2.191	0.802	0.897	0.384	2.095
Fifth	0.002§	3.365	1.573	7.196	0.375	1.530	0.597	3.917
Sixth	0.043§	3.235	1.036	10.104	0.870	1.117	0.297	4.195
Residence								
Urban*	-	-	-	-	-	-	-	-
Rural	0.003§	0.525	0.342	0.808	0.572	0.866	0.527	1.425
Daily sun exposure (hour)								
< 1 *	-	-	-	-	-	-	-	-
1–3	0.058	1.418	0.988	2.035	0.003§	1.900	1.248	2.892
> 3	0.352	0.832	0.565	1.226	0.194	1.358	0.856	2.156
Vitamin D level								
< Normal *	-	-	-	-	-	-	-	-
Within normal	0.172	0.704	0.426	1.165	0.622	1.158	0.647	2.072
> Normal	0.361	0.324	0.029	3.641	0.954	1.092	0.054	22.085
Do not know	< 0.001§	0.422	0.292	0.609	0.006§	0.557	0.368	0.844

Notes:

* reference value;

OR = odd ratio; AOR = adjusted odd ratio; CI = confidence interval;

§ *P* < 0.05
